# Molecular Evidence of *Sarcocystis* Species Infecting Reptiles in Peninsular Malaysia

**Published:** 2019

**Authors:** Nahdatul Fatihah MOHD FADIL, Tengku Idzzan Nadzirah TENGKU-IDRIS, Shahhaziq SHAHARI, Mun Yik FONG, Yee Ling LAU

**Affiliations:** 1. Department of Parasitology, Faculty of Medicine, University of Malaya, Kuala Lumpur, Malaysia; 2. Department of Diagnostic and Allied Health Science, Faculty of Health and Life Sciences, Management & Science University, Selangor, Malaysia

**Keywords:** *Sarcocystis*, Sarcocystosis, Snake, 18S rDNA, Phylogenetic analysis, Malaysia

## Abstract

**Background:**

The genus *Sarcocystis* consists of intracellular coccidian protozoan parasites with the ability to invade muscle tissue and mature into sarcocysts, causing the zoonotic disease sarcocystosis. These parasites have an obligatory two-host life cycle, which correlates with prey-predator relationship. The distribution and prevalence of *Sarcocystis* in reptiles remains unclear, despite several previous reports. The aim of this study was to identify the genetic assemblage of the species of *Sarcocystis* infecting Malaysian snakes and lizards by screening stool samples.

**Methods:**

Overall, 54 fecal samples of various snake species and four fecal samples of several lizard species in Malaysia were taken within the course of August 2015 to January 2016 from Seremban, Melaka, Tioman Island, Pahang, Klang and Langkawi Wildlife Park located in Malaysia. The samples were examined for *Sarcocystis* through PCR amplification of the 18S rDNA sequence at the Department of Parasitology, University of Malaya

**Results:**

Fourteen snake fecal samples were positive via PCR; however, only eight samples (14%) were found positive for *Sarcocystis* species, whereas four were positive for other genera and the identity of another three samples was unable to be determined. Further phylogenetic analysis of the 18S rDNA sequences revealed that the snakes were infected with either *S. singaporensis*, *S. lacertae*, or undefined *Sarcocystis* species closely related to either *S. singaporensis* or *S. zuoi*. *Sarcocystis nesbitti* infection was not identified in any of the infected snakes.

**Conclusion:**

This is the first report of identification of *S. lacertae* in the black-headed cat snake.

## Introduction

The life cycle of *Sarcocystis* species relies greatly on an obligatory prey-predator relationship between the intermediate and definitive hosts, in which the intermediate and definitive hosts vary for each species of *Sarcocystis*. Asexual development of *Sarcocystis* occurs in the intermediate host, which is usually the prey, and sexual development occurs in the definitive host, which is the predator. Definitive hosts are infected through ingestion of muscular of the intermediate host containing mature sarcocysts and undergo sexual development in the small intestine. Sporocysts are shed in the feces of the definitive host and will eventually infect the intermediate host through fecal contamination of food or water ingested by the intermediate host (1).

Humans serve as definitive hosts for two known *Sarcocystis* species, *S. hominis* and *S. suihominis*. *S. hominis* is acquired through ingestion of beef infected with sarcocysts, while *S. suihominis* is acquired through ingestion of pork infected with Sarcocystis, and both lead to intestinal sarcocystosis in humans. *S. suihominis* is more pathogenic that *S. hominis*, which is only mildly pathogenic for humans. However, humans may also serve as the intermediate host for several unidentified *Sarcocystis* species, including *S. nesbitti*, found to be involved in the biggest human muscular sarcocystosis outbreak recorded to date (1).

In 2012, GeoSentinel and TropNet reported cases of acute muscular sarcocystosis among travelers returned from Tioman Island between 2011 and 2012 (2). Eighty-nine cases were subsequently reported involving a group of college students and teachers following a retreat in Pangkor Island (3). Symptoms of muscular sarcocystosis include acute fever, myalgia, myositis, vasculitis, headache, cough, and diarrhoea, and some cases reported pruritic rash, bronchospasm, or oedema of the face or an extremity (1). Sarcocysts were identified through histological examination of three out of four muscle biopsies obtained from the temporalis, tibialis posterior, and two gastrocnemius muscles of the patients involved in the Pangkor Island outbreak (3) and in the muscle of six patients involved in the Tioman Island outbreak (4). Further molecular characterization of *Sarcocystis* species by detection of 18S rDNA sequences revealed *S. nesbitti* in patients from both Tioman (4) and Pangkor Island (3).

*S. nesbitti* was first described in the muscle tissue of rhesus monkeys *Macaca mulatta* (5) and crab-eating macaques *Macaca fascicularis*, which implied that nonhuman primates serve as an intermediate host (6). An estimated prevalence of 21% for muscular sarcocystosis in humans in Malaysia and an estimated prevalence between 0% and 3.6% in western countries have been reported based on postmortem examination (7). A higher prevalence was seen in Malaysia, nonhuman primates are more common in Malaysia in comparison to western countries (7).

In 2011, snakes might serve as a definitive host for *S. nesbitti* due to the fact that *S. nesbitti* has a close phylogenetic resemblance to other *Sarcocystis* species involved in the rodent-snake cycle, such as *S. zamani*, *S. zuoi,* and *S. singaporensis* (8). *S. nesbitti* was later found in the fecal samples of a reticulated python from Langkawi and a monocled cobra from Kelantan. Subsequent phylogenetic analysis confirmed that snakes are likely the definitive host for *S. nesbitti* (9). In 2013, a prevalence of 25% for *Sarcocystis* infection in the Malaysian snake population was reported. The water monitor lizard might be an alternative definitive host for *S. nesbitti*. This was suggested because, in comparison with snakes, monitor lizards are often sighted in close proximity to humans (10).

Based on this, a hypothesis was put forward that is a definitive host of *S. nesbitti* is not limited to snakes, but also to reptiles in general. The objective of this study was to identify the genetic assemblage of the species of *Sarcocystis* infecting Malaysian snakes and lizards by screening stool samples.

## Materials and Methods

### Sample collection

Fifty-four snakes and four lizard fecal samples were collected from various snake farms and reptile captors from Seremban, Melaka, Tioman Island, Pahang, Klang and Langkawi Wildlife Park located in Peninsular Malaysia within Aug 2015–Jan 2016. These include reticulated python (*Python reticulates*), albino reticulated python, bronzeback tree snake (*Dendrelaphis tristis*), black-headed cat snake (*Boiga nigriceps*), king cobra (*Ophiophagus hannah*), Burmese python (*P. bivittatus*), blood python (*P.brongersmai*), albino green Burmese python, ball python (*P. regius*), spitting cobra (*Naja* sp.), copperhead racer (*Coelognathus radiates*), man-grove snake (*B. dendrophila*), cave racer (*Othriophis taeniurus*), carpet python (*Morelia spilota*), oriental whip snake (*Ahaetulla prasina*), iguana (*Iguana iguana*), gecko (*Eublepharis macularius*) and monitor lizard (*Varanus salvator*).

This study was carried out with the approval by Institutional Animal Care and Use Committee (IACUC) of the University of Malaya, Faculty of Medicine (2015-180908/PARA/R/LYL).

Each sample was immersed in PBS containing 1000 units/mL of penicillin, 100 ug/mL of streptomycin and 2.5 ug/mL of fungizone antimycotic in stool containers. The collected samples were transported back to Department of Parasitology, Faculty of Medicine and stored in cold room at 4 °C for further use.

### DNA extraction

The sample was homogenized by vortexing and DNA extraction was performed on 0.25 g of the fecal sample according to the manufacturer’s protocol (PowerSoil® DNA Isolation Kit, MO Bio Laboratories). DNA was eluted with 100 μl of elution buffer and stored at 4 °C until processed.

### Polymerase chain reaction

Nested PCR for 18S rRNA was performed according to the previous published paper. ^9^ The primers involved were 1H (Forward: 5′CCA TGC ATG TCT AAG TAT AAG C 3′) and 1L (Reverse: 5′ AAC TGT TAT TGC CTC AAA CTT C 3′) for Nested 1 and 2H (Forward: 5′ CTA GTG ATT GGA ATG ATG GG 3′) and 3L (Reverse: 5′ AAC TGT TAT TGC CTC AAA CTTC 3′) for Nested 2. ^6^ PCR amplification was carried out in a 25 μl mixture containing 35 mM Tris–HCl, pH 9.0, 25 mM KCl, 3.5 mM MgCl _2_ , 5 pmoles of each primer, 1 mM dNTPs, and 1 U Taq polymerase (Promega) and 4 μl of DNA template. The mixture was subjected to PCR condition as follow: 94 °C for 2 min and 35 cycles of denaturation for 40 sec at 94 °C, annealing for 30 sec at 50 °C and 1.5 min extension at 72 °C. A final extension of 10 min at 72 °C was added at the last cycle. Expected size of the amplicon was approximately 1000 base pairs. The PCR product was viewed on 1% (w/v) aga-rose gel.

### Cloning and sequencing

Each PCR product was cloned into pGEM®-T Vector System (Promega) and positive clones were selected for bi-directional sequencing using M13 universal primers. Sequencing results from MyTACG Bioscience Enterprise were analyzed using the BioEdit software.

### Phylogenetic analysis

Sequencing results from MyTACG Bioscience Enterprise were analyzed using the BioEdit software. Two to three sequences from the same amplicon were aligned using ClustalW before similarity searches were carried out using Basic Local Alignment Search Tool (BLAST) to identify the match based on the 18S rDNA sequences. Phylogenetic tree was constructed using Neighbor-Joining method (bootstrap=1000) available in MEGA 6 software. The 18S rRNA gene sequence of *Eimeria tennella* was used as outgroup.

## Results

**Fig. 1:**
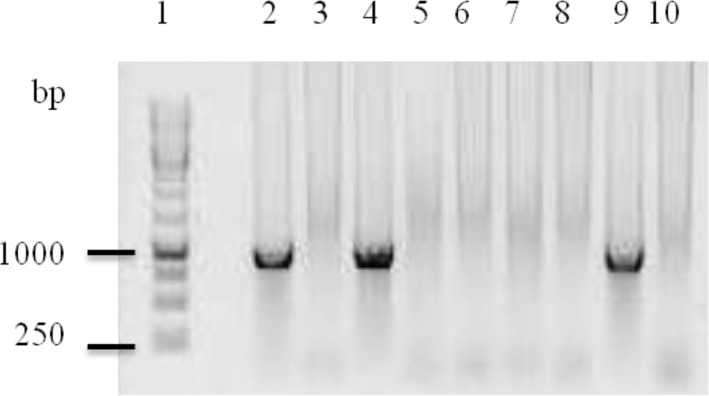
Agarose gel electrophoresis of 18S rDNA gene. Positive PCR of 18S rDNA gene generated a single band with the expected size of 1000 bp (lanes 2 and 4). Lane 9 serves as positive control. No band was observed for non-template negative control (lane 10) and negative PCR samples (lanes 3,5,6,7 and 8). Lane 1 serves as 1kb DNA ladder

Out of the 54 snake, fecal samples and four lizard fecal samples collected, 15 snake fecal samples were found positive for *Sarcocystis* 18S rDNA and none of the lizard fecal samples were found positive based on the PCR results (Fig. 1).

Running sequencing results through the BLAST database revealed that eight snakes (14%) were infected with *Sarcocystis* species (Table 1).

**Table 1:** Summary of *Sarcocystis* species found in infected reptiles

***Reptile taxa species***	***Provenance***	***Number of positive reptile***	***Clone name***	***Sarcocystis species***
Reticulated python (*P. reticulatus*)	Klang (KLA)	3	KLA005(2), KLA008(1), KLA008(2), KLA010(1)	*S. singaporensis* (AF434057)
			KLA005(3)	*S. singaporensis* (AF434051)
			TIO203(1)	*S. singaporensis* (KC878481)
			TIO203(2)	*Sarcocystis* sp. (KC201640)
			TIO203(3)	*S. singaporensis* (AF434054)
			TIO201(3), TIO201(4), TIO202(4), TIO202(5)	*Sarcocystis* sp. (KC878486)
			TIO202(3)	*Sarcocystis* sp. (KC878480)
King cobra *O. hannah*)	Seremban (SER)	1	SER007(1), SER007(4)	*Sarcocystis* sp. (AB691780)
Black-headed cat snake (*B. nigriceps*)	Tioman Island (TIO)	1	TIO205(4)	*S. lacertae* (AY015113)

Six of these snakes were reticulated pythons (*P. reticulates)*, three from Klang and three from Tioman Island, one was a king cobra (*O. hannah)* from Seremban, and the last was a black-headed cat snake (*B. nigriceps)* from Tioman Island.

**Fig. 2:**
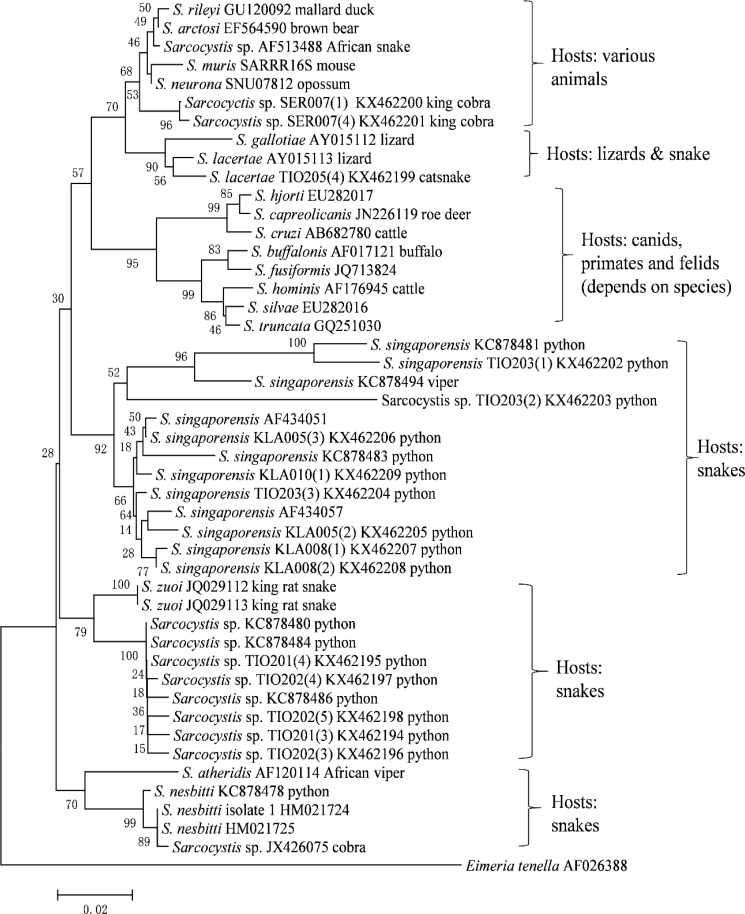
Phylogenetic tree based on 18S rRNA sequences of *Sarcocystis* species with plotted final hosts

All of these snakes were wild caught and their diet in captivity consisted of mostly lizards, rodents, or chickens. The other four fecal samples found positive for *Sarcocystis* 18S rDNA contained *E. cahirinensis, Caryospora bigenetica*, *Spumella* spp., and *E. tenella*, while another three positive results were unable to be determined due to poor sequencing results. A phylogenetic tree was constructed using MEGA 6 software. The 18S rDNA sequences of the samples were compared to thirty-one *Sarcocystis* species sequences obtained from Genbank, with *E. tenella* as outgroup. The strength and reliability of the phylogenetic tree were assessed using bootstrap analysis with 1000 replications, where a bootstrap value of 70% was considered as strongly supported, 50% to 70% as moderately supported and below 50% as weakly supported. Neighbor-Joining method modeling was used to construct the phylogenetic tree, which showed six groups based on the respective definitive hosts of the different *Sarcocystis* species (Fig. 2). The first group consisted of species whose hosts are various animals, including mallard duck, opossum, and mouse; the second group consisted of species that infect lizards and snakes; and the third group consisted of species that infect canids, cats, and humans. Interestingly, the fourth, fifth, and sixth groups consisted of species that use snakes as definitive hosts. The fourth group was made of mainly *S. singaporensis*, the fifth contained *S. zuoi* and unidentified *Sarcocystis* spp., and the sixth group consisted of mainly *S. nesbitti*. The *Sarcocystis* species detected in snake fecal samples from this study were distributed among all of the groups, except in the last group with *S. nesbitti*. The bootstrap values for the branches of each group ranged from 73% to 99%, which means that the tree is strongly supported. Data for this project was deposited into National Center for Biotechnology Information (NCBI) under the accession numbers KX462194-KX462209 and are available through GenBank at (https://www.ncbi.nlm.nih.gov/genbank/).

## Discussion

Eight out of fifteen snake fecal samples were found to be infected with *Sarcocystis* species by DNA sequencing of 18S rDNA. All of these snakes were caught in the wild and their diet in captivity consisted of mostly lizards, rodents, or chicks. This gave us information on the possible lifecycle of *Sarcocystis* species, the diets of these snakes were similar in the wild and in captivity. However, other information such as the length of captivity and the origin of the snake were not provided by the owner. The limited samples obtained vary throughout the various geographical locations as samples collection was based on the animal owners’ verbal agreement to participate in the study.

Another four positive results were identified via sequencing as *E. cahirinensis*, *C. bigenetica*, *Spumella* spp., and *E. tenella*. These organisms, like *Sarcocystis*, are apicomplexan and so possess the 18S rDNA gene. The 18S rDNA gene encodes the structural RNA for the small component of eukaryotic cytoplasmic ribosomes and 18S rDNA of other closely-related genera have similar sequences. Thus, the 18s rDNA of other genera was amplified during nested PCR. This might also occur due to un-specific binding of the primers. Although other genes, such as cytochrome oxidase 1 (COX1) and internal transcribed spacer (ITS), can also be used for species-specific identification, there are less sequencing data and information on these genes available for differentiating *Sarcocystis* species and carrying out phylogenetic modeling (7). Diagnosis of *Sarcocystis* sp. is generally by done by microscopic examination of the sporocysts in feces.

Microscopic examination was attempted on few samples but remained negative for oocyst and sporocysts. Microscopy examination has low sensitivity and cannot differentiate between species (11).

Hence, sequencing of 18S rDNA appears to be the best method for species differentiation within *Sarcocystis*. Lizards are commonly established as the intermediate host in heteroxenous life cycles of *Sarcocystis* species (12, 13) which might explain the absence of *Sarcocystis* in stool sample of lizards in this study.

Lizards only serve as both intermediate and definitive host in the monoxenous life cycle of certain *Sarcocystis* species, such as *S. gallotiae*. In a monoxenous life cycle, *Sarcocystis* species are transmitted by cannibalistic behaviors among lizards, in which they feed on their own species (14). Sarcocysts and oocysts of *Sarcocystis* species can be found in the muscular tissue of the tail and in the intestine of lizards, respectively (15). These cannibalistic behaviors were not seen in the lizards involved in this study, as they were singly isolated in their captivity.

All of the python fecal samples found positive for *S. singaporensis* were grouped together in the phylogenetic tree with the same species obtained from GenBank. The range of definitive hosts for *S. singaporensis* was limited to the snake family Boidae, which includes reticulated pythons (16) and that *S. singaporensis* is known to use the reticulated python as a definitive host (12). The known intermediate hosts for *S. singaporensis* include all *Rattus* and *Bandicota* species and *Nesokia indica* (16).

This is the first identification of *S. lacertae* in the black-headed cat snake. *S. lacertae* was originally described as having a heteroxenous life cycle that combined two reptilian hosts, the common wall lizard (*Podarcis muralis*) as the intermediate host and the colubrid snake (*Coronella austriaca*) as the definitive host (17). The black-headed cat snake and the colubrid snake belong to the same family, Colubridae, which suggests that they share a similar diet and thus also share the possibility of being infected with *S. lacertae*. Three reticulated pythons from Tioman Island and a king cobra from Seremban were found positive for unidentified *Sarcocystis* spp., and one of these reticulated pythons from Tioman Island (TIO203) was infected with both the unidentified *Sarcocystis* species and *S. singaporensis*. These unidentified *Sarcocystis* species might be related to each other and to *S. zuoi*, as all of them were included in the same group, with the exception of the *Sarcocystis* spp. involved in the double infection of TIO203, which instead showed close relation to *S. singaporensis*.

In this study, *S. nesbitti* infection was not identified in any of the infected reptiles. *S. nesbitti* infection was discovered in a reticulated python from Langkawi Wildlife Park, but samples collected from the exact same snake yielded negative results in this study. This might have occurred because the samples were not collected directly by fresh fecal swabbing from the snake of interest, but instead dried fecal samples were collected from the cage that the infected snake shared with another reticulated python. This might also have occurred due to a complete shed off of *Sarcocystis* infection in the snake.

The phylogenetic tree constructed in this study supports the findings of previous studies which established that the phylogenetic relationships among the known *Sarcocystis* species suggested their coevolution with their definitive hosts, rather than their intermediate hosts (9, 18). The *Sarcocystis* species isolated from snakes in this study are related to *S. singaporensis*, *S. zuoi*, and *S. lacerate*, which also use snakes as their definitive hosts. The bootstrap method was used to assess the reliability of the phylogenetic tree constructed, although the percentages shown in this study were not as high as those found.

## Conclusion

This study presents the latest prevalence (14%) of *Sarcocystis* infection in snake and lizard populations in Peninsular Malaysia. Different species of snakes were found to be infected with *S. singaporensis*, *S. lacerate*, and some undefined *Sarcocystis* species. *S. nesbitti* infection was not identified in any of the infected snakes. For further study, a greater number of samples from different species of snakes and monitor lizards, specifically, need to be obtained in order to accept or reject the hypothesis that definitive hosts of *S. nesbitti* are restricted not just to snakes but to reptiles in general. In addition, a more stringent phylogenetic approach should be used to construct a reliable phylogenetic tree.
